# Manipulation of Convection Using Infrared Light Emitted from Human Hands

**DOI:** 10.1002/advs.202307020

**Published:** 2024-01-18

**Authors:** Hanrui Zhu, Zhen Luo, Lifu Zhang, Qingchen Shen, Runheng Yang, Weizheng Cheng, Yingyue Zhang, Modi Jiang, Chunzhi Guo, Benwei Fu, Chengyi Song, Peng Tao, Shun An, Wen Shang, Tao Deng

**Affiliations:** ^1^ State Key Laboratory of Metal Matrix Composites School of Materials Science and Engineering Shanghai Jiao Tong University Shanghai 200240 P. R. China; ^2^ Department of Materials Science and Engineering Rensselaer Polytechnic Institute Troy NY 12180–3590 USA; ^3^ Yusuf Hamied Department of Chemistry University of Cambridge Cambridge CB2 1EW UK; ^4^ School of Electronic Information and Electrical Engineering Shanghai Jiao Tong University Shanghai 200240 P. R. China; ^5^ Shanghai Key Laboratory of Hydrogen Science School of Materials Science and Engineering Shanghai Jiao Tong University Shanghai 200240 P. R. China

**Keywords:** convection controlling system, human hand, infrared radiation

## Abstract

Control of convection plays an important role in heat transfer regulation, bio/chemical sensing, phase separation, etc. Current convection controlling systems generally depend on engineered energy sources to drive and manipulate the convection, which brings additional energy consumption into the system. Here the use of human hand as a natural and sustainable infrared (IR) radiation source for the manipulation of liquid convection is demonstrated. The fluid can sense the change of the relative position or the shape of the hand with the formation of different convection patterns. Besides the generation of static complex patterns, dynamic manipulation of convections can also be realized via moving of hand or finger. The use of such sustainable convections to control the movement of a floating “boat” is further achieved. The use of human hands as the natural energy sources provides a promising approach for the manipulation of liquid convection without the need of extra external energy, which may be further utilized for low‐cost and intelligent bio/chemical sensing and separation.

## Graphical abstract

In this work, the control of convection is demonstrated using the human hand as a natural IR radiation source. Both static and dynamic manipulation of convection can be realized with the use of such IR light source. This study also demonstrates the use of the generated convection for the controlled movement of a floating “boat.”

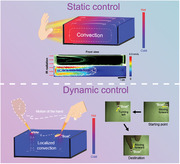

## 1 Introduction

Convection commonly occurs in nature, such as in the atmosphere,^[^ ^1^ ^]^ in the ocean,^[^ ^2^, ^3^, ^4^ ^]^ in ice shelves,^[^ ^5^ ^]^ and around the sun.^[^ ^6^, ^7^ ^]^ Convection most of the time leads to the energy exchange and the mass transfer, and is widely used in many different systems,^[^ ^8^, ^9^, ^10^ ^]^ including motion systems^[^ ^11^, ^12^ ^]^ and heat transfer systems.^[^ ^13^, ^14^ ^]^ Convection can also carry specific nanoparticles or targeting molecules for directional transportation, which further broadens the application of convection in bio/chemical sensing systems^[^ ^15^, ^16^ ^]^ and separation systems.^[^ ^17^ ^]^ Generally, the formation of the convection is the result of the nonequilibrium state inside the liquid.^[^ ^18^, ^19^ ^]^ One type of the nonequilibrium state is the temperature gradient that is usually generated by engineered energy sources, including the hot plate,^[^ ^20^, ^21^ ^]^ the IR lasers,^[^ ^22^, ^23^ ^]^ the visible lamp,^[^ ^24^ ^]^ and the ultraviolet (UV) light source.^[^ ^25^ ^]^

The engineered energy sources for generating and controlling the convection can be simply classified as contact sources^[^ ^26^, ^27^ ^]^ and non‐contact sources.^[^ ^28^, ^29^, ^30^ ^]^ The use of contact energy sources may lead to the mechanical wear and fatigue,^[^ ^31^ ^]^ and the contamination of the fluid may also happen through the direct‐touching operation. Therefore, non‐contact energy sources, with the advantages of reduced mechanical wear and minimized contamination of fluids, are gradually involved in many convection systems.^[^ ^32^, ^33^, ^34^ ^]^ The man‐made non‐contact energy sources, however, require the use of external energy for their proper operation, which may limit their potential use. For example, Zhang et al. investigated an approach to manipulate the in‐fiber nanoparticles by inducing thermocapillary convection via a far‐IR laser with the maximum power of 20 W.^[^ ^35^ ^]^ The approach is interesting, but the need of using high‐power energy sources, however, may limit the application scope of this method. The exploration of alternative non‐contact energy sources that do not need energy supply may broaden the applications of the convection systems.

Compared with those engineered energy sources, natural energy sources have the advantages of zero extra energy consumption and sustainability.^[^ ^36^ ^]^ Currently various natural energy sources have been broadly used in many engineered systems such as photovoltaic,^[^ ^37^, ^38^ ^]^ seawater desalination,^[^ ^39^ ^]^ and interfacial evaporation.^[^ ^40^ ^]^ Human hands, with relatively stable temperature and the ability of continuously emitting IR light, have been demonstrated as a new type of intelligent and sustainable natural energy source.^[^ ^41^, ^42^, ^43^ ^]^ An et al. have reported that human hand can be considered as natural IR light source to interact with the structured surfaces and the generated signals can be further used for sign language recognition^[^ ^41^ ^]^ and human‐machine interaction.^[^ ^43^ ^]^ The integration of human hands into engineered systems brings the advantages of sustainability and intelligent controllability into these systems. Therefore, the integration of human hands into the convection systems as the non‐contact energy source can not only reduce the energy consumption but also improve the intelligence of the convection systems.

**Figure 1:**
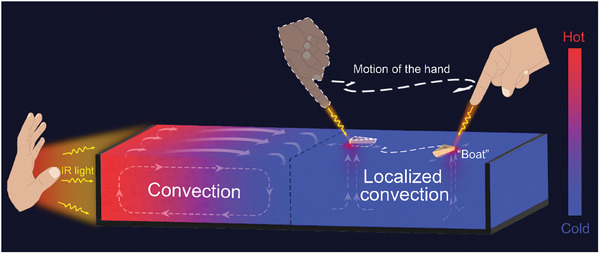
Schematic illustration of the controllable convection generated by human hands. The IR radiation from human hands enables the generation of the temperature gradient within the liquid, which induces convections in the liquid. The localized convections can be generated both statically and dynamically and also can be manipulated by hands. With controllable localized convection, the floating “boat” can be touchlessly manipulated by human hands.

In this work, we demonstrate the convection in the fluidic system could be generated and controlled by sensing the IR radiation from the human hands. The use of human hands enables the formation of multiple complex convection patterns as well as the control of those convections. The control of floating “boat” through hand enabled convection is also demonstrated. As shown in **Figure** **1**, the fluidic system sensed the temperature difference induced by the hand‐emitted IR radiation and thus convection was generated. With the change of the positions of hands, convections can be sustainably generated at different locations, which can be further used to control the movement of the floating “boat” in real‐time. In the study, we used the polystyrene (PS) nanoparticles to trace the flow in the convection and investigated the influence factors that affect the velocity field and the lateral size of the convection. The results show that the velocity and the lateral size increase as the depth of the water gets larger and the hands get closer to the container. Different patterns of convections can be generated both statically and dynamically with the use of human hands. With the established manipulation of the convection through hands, we further achieved the control of the motion of a floating “boat” at the liquid‐air interface. The use of human hands as the IR energy sources offers a powerless and non‐contact approach for the generation and control of the mass transfer in the convection at will, which may further broaden the potential applications of convection in low‐cost sensing and separation systems.

## 2 Results

### 2.1 Generation of Single Convection Using Human Hands

**Figure 2:**
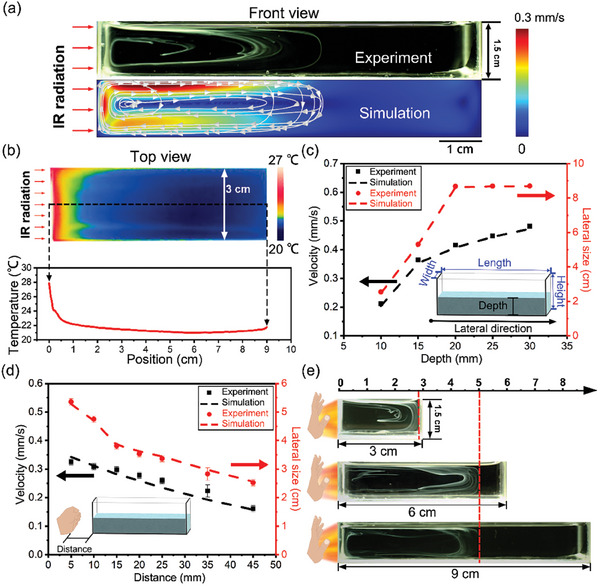
The generation of the convection by hand. a) The experimental observation of the convection generated by placing one hand on the left side of the container (top) and the simulated velocity field of the convection (bottom). b) The IR image (top view) of the container shows the temperature distribution in the water. The plot underneath the IR image shows the temperature along the dotted line across the IR image. c,d) The change of velocity and lateral size of the convection with the change of c) the depth of the water and (d) the distance between the hand and the container. The error bars represent the standard deviation (SD) of the mean (n = 3) e) The optical images of convection patterns formed in the containers with different lengths that are induced by the IR radiation from the hand.

**Figure 3:**
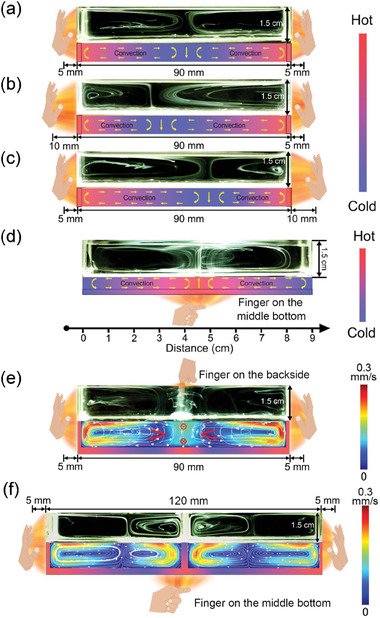
Static formation of the convections with complex patterns. a–c) By controlling the distance between hands and the sidewalls of the container, convections can be formed both a) symmetrically and b,c) asymmetrically. d) The generation of two symmetric convection patterns by placing a finger under the middle bottom of the container. e) Using two hands and one finger to form three convection patterns (on the right, left, and backside of the container) at the same time. f) Four convection patterns can be generated symmetrically by placing hands and the finger on the right, left, and bottom of the container at the same time, with a quartz plate inserted in the middle of the container.

**Figure 4:**
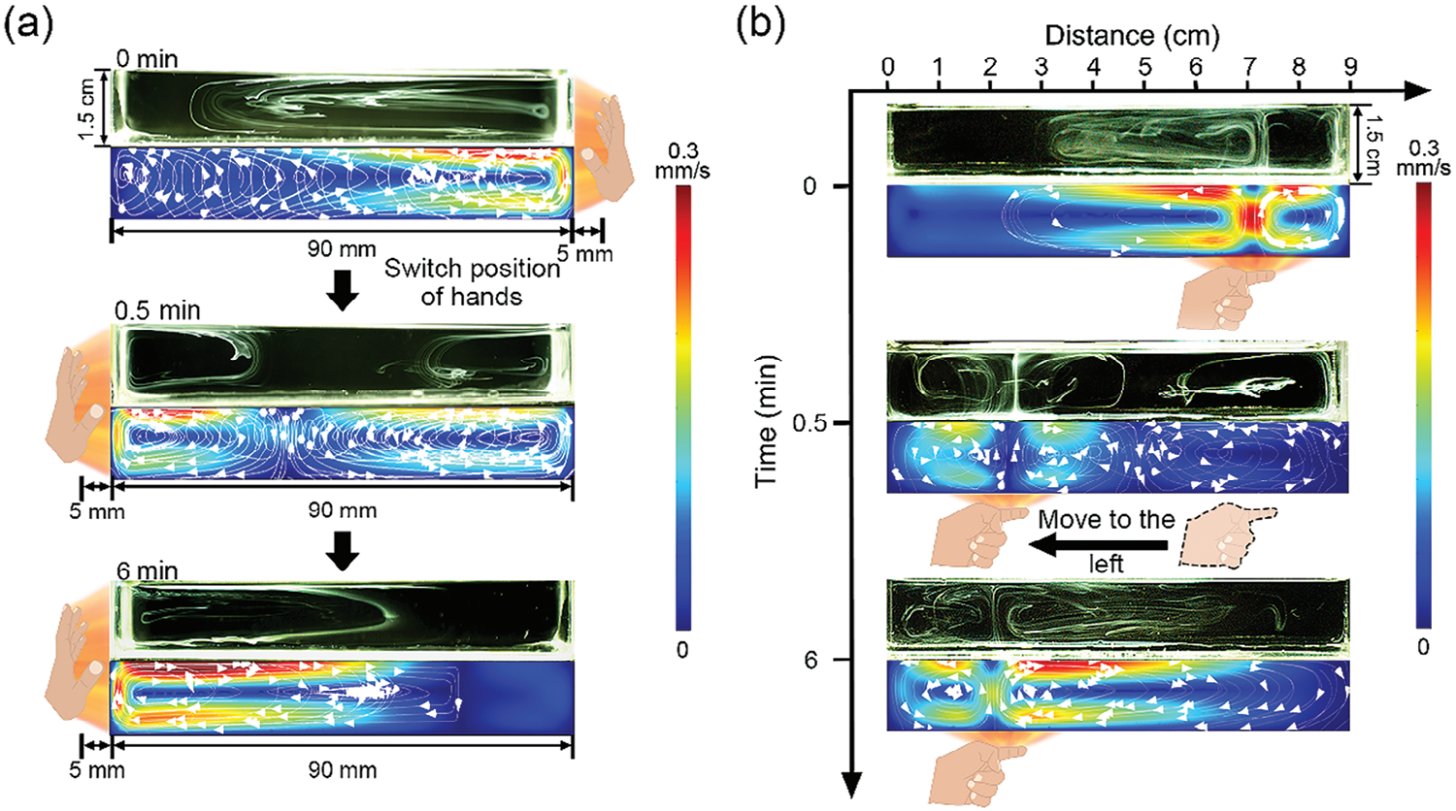
Dynamic control of the convection. a) The convection can be controlled dynamically by switching the hand position to different sides of the container. Numerical simulation shows the change of the velocity field during this process. b) With the moving of the finger position below the bottom surface of the container, the convections move with the moving of finger. Numerical simulation shows the change of velocity field during this process.

The human hand can be considered as an incoherent IR energy source with a temperature of ≈310 K and an emissivity of ≈0.98.^[^ ^44^ ^]^ Based on the Blackbody radiation law, the wavelength of the IR radiation of the hands mainly concentrates in the range of 4–16 µm. In this study, we explored the use of the hand as a natural IR energy source for the generation and manipulation of convections in liquid. The experimental setup is shown in Figure S1a (Supporting Information), which includes a high‐speed camera and a quartz container half‐filled with deionized (DI) water. In order to be consistent in using the same amount of the IR radiation from the hand, we used the aluminum foil to cover the palm of the hand with an open emission window of 4 cm × 4 cm (≈ 0.83 W). The size of the open window is larger than the size of the sidewall of the container (Figure S1b, Supporting Information). The PS nanoparticles (Invitrogen Trading Co., Ltd) with average size of ≈ 206 nm (Figure S2, Supporting Information) were used to trace the movement of liquid flows. As shown in Figure S3 (Supporting Information), both the DI water and the quartz can absorb the IR radiation.^[^ ^45^ ^]^ During the experiment, the hand was placed parallel to the left sidewall of the container with a distance of 5 mm from the container. The IR radiation from the hand can be absorbed by the left side of the container half‐filled with water, which leads to the temperature difference within the liquid, and subsequently induces the convection of the liquid (**Figure** **2**a) and Movie S1, Supporting Information). The response time, the time from placing hand near the container to the time that the PS particles start to move, was measured to be ≈ 5 s. The time that the PS particles complete one cycle of convection is obviously longer than the response time and is 6 min, which can be defined as the cycle time. In Figure 2a and the following experiments (Figures 2, 3, 4), we placed the hand close to the container for one cycle and captured the image of the convection at the same time, aiming to visualize the complete cycle of the convection for further characterization.

Numerical calculation was further taken to simulate the formation of the convection. The input power on the left side of the container is the IR radiation emitted from hands (Section S1, Supporting Information). With the known input energy, the velocity field and the temperature field of the fluid can be obtained by using COMSOL Multiphysics (Section S2, Supporting Information) to solve the Navier‐Stokes equations. In this study, the temperature‐induced density change of the liquid is much less than the density of the liquid, we thus used the Boussinesq approximation (Section S3, Supporting Information)^[^ ^46^ ^]^ to simplify the Navier‐Stokes equations. The simplified equations can be given as:

(1) ∂ρ0∂t+∇·ρ0u=0ρ0∂u∂t+ρ0u·∇u=−∇p+∇μ∇u+∇uT−23μ∇·uIρ0∂u∂t+ρ0u·∇u=+Fρ0Cp∂T∂t+ρ0Cpu·∇T=∇·k∇T+Q

where ***u***, *Q*, *p*, and ***F*** are the velocity vector, the internal heat source of the fluid, the approximated pressure (Boussinesq approximation, Section S3, Supporting Information) and the approximated body force (Boussinesq approximation, Section S3, Supporting Information), respectively. **I** is the identity matrix and *C_p_* and *k* are the heat capacity at constant pressure and the thermal conductivity of the liquid, respectively. The approximated body force by Boussinesq approximation ***F *** =− ρ_0_β(*T* − *T*_0_) ***g***, in which ρ_0_ is the initial density of the liquid at the initial temperature *T*_0_ and β is the coefficient of thermal expansion of the fluid at *T*_0_. For the original state without hand, the liquid is in temperature equilibrium with the environment, which means *T* − *T*_0_ =  0, thus ***F*** equals to **0**. For the condition of placing hands close to the container, the IR radiation from hand would lead to the temperature increase of the liquid. In this case, *T* − *T*_0_ ≠ 0, so ***F*** ≠ 0, and there is coupling between density and temperature. With the temperature‐induced buoyancy force, the convection could be generated. Figure 2a shows that the simulating results are in good agreement with the experimental results. We also ran a control experiment to prove that the convection was induced by the temperature change (Figure S4, Supporting Information). Without putting hand on the left side of the container, the liquid and the container can only receive the IR radiation from ambient. Since the ambient has the same temperature with the liquid, the IR radiation from the ambient would not cause the change of temperature in the liquid and thus the convection was not formed in the fluid.

An IR camera (FLIR T640) was used to visualize the temperature distribution of the water when hand was placed close to the left side of the container. Both Figure 2b and Figure S5 (Supporting Information) show that the temperature of the left side of the container is higher than that of the right side. We also explored the effect of environmental temperature and movements of extra IR light sources on the convection. As shown in Figure S6 (Supporting Information), the velocity and the lateral size of the convection, which is the length of convection along the lateral direction, decreased with the increase of the environmental temperature due to the smaller temperature difference within the fluid. Under different environmental temperatures, the convection keeps stable, with the standard deviation (SD) of the lateral size and the velocity in all experiments about 0.06 cm and 0.004 mms^−1^, respectively. To explore the effect of the movement of an extra IR light source, we placed one hand on the left side of the container to generate a stable convection. We then controlled another hand moving behind the container with a speed of 1.2 cm s^−1^ and the distance to the container of 5 cm. As shown in Figure S6b (Supporting Information), the lateral size and the velocity of the convection did not change with the movement of the extra IR light source (another hand). The convection pattern was stable, with little interference from the moving hand.

We further analyzed the factors that influence the generation of the convection, including the depth of the liquid, the distance between the hand and the container, and the length of the container. We first investigated the effect of the depth of the DI water on the velocity and the lateral size of the convection. Despite the penetration depth of IR radiation in liquid water is less than 100 µm (Section S4, Supporting Information), the lateral size of the convection flow is in the centimeter scale since the temperature difference would exist in a much longer distance in the medium due to the thermal conduction. As shown in Figure 2c, the velocity of the convection (Experimental Section) increased with the increase of the water depth from 10 to 30 mm in the container with 9 cm length. The lateral size of convection also became larger with the increase of the depth of the water. When the depth of water was over 20 mm, the convection circle was confined by the container and thus the lateral size of the convection was all ≈ 9 cm. Therefore, in order to ensure the formation of the convection circle completely, the depth of the water was set as 15 mm in most of the experiments in this study. The other factor that affects the convection is the distance between the hand and the container. As shown in Figure 2d, the velocity and the lateral size of the convection decreased with the increase of the distance between the hand and the container. In the study we set the distance between the hand and the container as 5 mm to obtain clear convection patterns. We further used COMSOL to numerically calculate the change of the convection properties with the change of water depth and distance between the hand and the container. As shown in Figure 2c,d, the calculated results of the convection are in good agreement with the experimental results.

We explored the effect of the thermal absorption characteristics of the container and the liquid medium on the effective working distance range by COMSOL simulation. As shown in Figure S7 (Supporting Information), the hand would not induce any convection if both the container and the liquid are non‐absorbing and thus there is no effective working distance in this scenario. For the fully IR‐absorbing container and non‐absorbing liquid, the convection still exists when the hand is 110 mm away from the container, and the effective working distance range is thus from 0 to 110 mm. Compared to fully IR‐absorbing container and non‐absorbing liquid, the system with non‐absorbing container and fully‐absorbing liquid always forms convections with larger lateral sizes and the effective working distance range is also larger, which is 0–125 mm, since the IR radiation could be directly absorbed by the liquid, with less heat loss by the wall of the container.

From Figure 2c, we found that the lateral size of the convection was limited by the available length of the container, which is the distance between two sidewalls. We further studied the effect of the length of the container on the lateral size of the convection (Figure 2e). In this experiment, the liquid depth was kept at 15 mm and the distance between hand and container was 5 mm. When the hand was placed at the left side of the containers, clockwise convections were stably formed in all three containers with different lengths (3 cm, 6 cm, and 9 cm). When the length of the container was small, the lateral size of the convection was limited by the length of the container. When the length of the containers was larger, the lateral size of the convections was not limited by the length of the container and all showed similar size of ≈5 cm. These results are consistent with the theoretical study by Ganzarolli and Milanez.^[^ ^47^ ^]^ The accuracy and sensitivity of the fluid velocity changes with energy are the crucial physical parameters of the convection systems (Section S5 and Figure S8, Supporting Information). In the unconstrained system (9 cm container), the accuracy was calculated to be 0.03 mm s^−1^ and the sensitivity was 0.441 mm s^−1^ W^−1^. Under different input energy, the generated convection also shows high stability with the SD of ≈ 0.003 mm s^−1^. In the constrained system (3 cm container), the stability, accuracy, and sensitivity were calculated to be 0.002, 0.025, and 0.344 mm s^−1^ W^−1^, respectively. Compared to unconstrained system, the constrained system has the similar accuracy and stability but the lower sensitivity of velocity change with energy, which might be due to the smaller temperature difference in shorter container. In the following experimental study, we chose the containers with lengths of 9 cm or larger to avoid the limitation of the length of the container.

### 2.2 Static Generation of Complex Convection Patterns Via Hands

With the demonstration of the generation of convection inside the fluid by the IR radiation from the hand, we further explore the use of hand to generate convections with different complex patterns. We first tried using two hands on both left and right sides of the container to generate convections (**Figure** **3**a–c). With the symmetric placement of hands on both left and right sides of the container, the input energy from both sides was the same, so two convection patterns with symmetric shapes were formed (Figure 3a). By controlling the distance between the hands and the sidewalls of the container, the two convections with asymmetric shapes can also be formed. As shown in Figure 3b,c, when we place two hands on both sides of the container with different distances, the input thermal energy is different between the left side wall and the right side wall of the container. The velocity and the lateral size of the convection decreased with the increasing distance between the hands and the container. Thus, if the distance between the hand and the sidewall on the left was larger than that on the right, the convection generated on the left side of the container would be smaller than that on the right side. Similar experimental results were also obtained when the distance between the right hand and the container was larger than that of the left hand. In ideal situation, the convection pattern in Figure 3b,c should mirror each other. In real experiments, however, the two convection patterns and also other convection pattern pairs did not mirror each other due to the possible variations in experiments including the slight variation of the dropped tracing solution (solution of PS nanoparticles), the slight variation of the relative position and angle of the hand, and the slight variation of the environmental and hand temperature. All of these factors may contribute to the variation of the convection patterns generated. We also used COMSOL to simulate the formation of the convections and the results are consistent with the experimental results (Figure S9, Supporting Information).

The IR light emitted from the hand should be generated not only by the palm of the hand but also by individual finger. We further explored the formation of the convections with different patterns inside the container by placing the index finger at different positions underneath the bottom surface of the containers (Figure 3d and Figure S10, Supporting Information). The distance between the index finger and the bottom surface of the container was still set as 5 mm. We first placed the index finger at the center of bottom surface of the container (Figure 3d). In such condition, the patterns of the convections were formed symmetrically in the container since the temperature difference was the same for both sides. The simulating result of the velocity field of the convection is consistent with the experimental result (Figure S11, Supporting Information). When we placed the index finger underneath the left bottom surface of the container, the convection was formed asymmetrically. The velocity and the lateral size of the convection on the left side of the containers was smaller than those on the right side (Figure S10i, Supporting Information). Such phenomenon can be attributed to two key factors. The first one is the temperature difference. According to Equation S5 (Supporting Information), the received radiation from fingers at different positions of the bottom surface of container is related to the distance between the finger and the position. When the finger is placed at the left side underneath of the container, the left far end of the bottom surface of the container receives more radiation than that on the right far end. Thus, the temperature difference within the right side of the container is greater than that at the left side. The velocity and the lateral size of the convection on the right side thus is larger than that of the left side. The other factor is the distance between the finger (warm side) and the sidewall (cold side). The convection on the left was confined by the distance between the finger and the left sidewall and same as the convection on the right of the container. The simulating results of the velocity field of the fluid inside the container are consistent with the above analysis (Figure S12, Supporting Information). Similarly, when we placed the index finger under the right side of the bottom of the container (Figure S10ii, Supporting Information), the convection was also formed asymmetrically and the only difference was that the velocity and the lateral size of the left convection in the container was larger than those of the right convection. The simulating results are also consistent with experimental results (Figure S12, Supporting Information).

We also explored the generation of complex convection patterns by placing multiple hands or fingers at different positions close to the container sidewalls. As shown in Figure 3e, when we placed the hands and the finger on the left, right, and backside of a 9 cm container, the convection was formed not only along the lateral direction but also the longitudinal direction. With the same distance of 5 mm between the energy source and the container, the energy input from the hand and the finger are apparently different, due to the larger surface area of the hand than that of the finger. The velocity of the convection formed on the backside was smaller than that of the convections on the left side and right side, which is attributed to the less energy input from the finger. Moreover, more complex pattern of four circulated convections was also generated by hands (and fingers) in a 12 cm container with a quartz plate inserted in the middle (Figure 3f). The convection pattern formed symmetrically due to the symmetrical energy input. By using just one finger as the energy source under the center of the bottom surface of the container, the energy input on the inserted quartz plate at the center of the container is smaller than the energy input on the left and right sidewalls of the container from the palm of the hands. The lateral sizes of both convections formed in the middle were thus smaller than those of the convections on the left and right. We also used the COMSOL to numerically calculate the velocity field and the results are in good agreement with the experimental results (Figure 3e,f).

### 2.3 Dynamic Manipulation of Complex Convection Patterns Via Hands

Besides the static generation of convections by hands, we also designed the following experiments to achieve dynamic control of the convections by hands and fingers. As shown in **Figure** **4**a, we first placed the hand on the right side of the container with the distance of 5 mm. After the convection stabilized, we then switched the hand to the left side of the container. At the beginning the IR radiation was absorbed by the right side of the container and the convection was stably formed on the right. After switching the hand to the left, the IR radiation was absorbed by the left side of the container. The previous temperature gradient on the right gradually decreased and the new temperature gradient established on the left. Consequently, the new convection began to generate on the left while the lateral size and the velocity of the convection on the right side gradually decreased. The right convection still existed for a few minutes, which was due to the residual heat from the right sidewall. The response time in the dynamic control of the convection, which is the time from the positioning of the light source at the new position to the starting of the formation of the new convection pattern, is ≈ 5 s, which is similar to the response time of the static generation of the convection. The stable convection was formed on the left after placing the hand on the left position for ≈ 6 min.

Similarly, dynamically control of the convections was also achieved by using just one finger, as shown in Figure 4b. We first placed the index finger under the right side of the bottom surface of the container with a distance of 2 cm. After the convection stabilized, we then moved the index finger to the left side of the bottom surface of the container (Figure 4b). The convection patterns also showed dynamic change that corresponded to the change of the finger position. When the finger was on the right side, two asymmetric convection patterns formed in the container. After moving the finger to the left side, the convection pattern changed. As shown in the middle picture in Figure 4b, before the system reached the steady state, there were three convections coexisted in the container, including two convections newly generated on the left and one on the right due to the residual heat. The convections became stable after placing the index fingers on the left bottom for 6 min. We also used COMSOL Multiphysics to simulate the change of the convection with the dynamic change of the hand/finger position. For such dynamic changes of the convection, transient simulation was used to investigate the intermediate state of the processes. As shown in Figure 4a,b, both simulation results were in good agreement with the results of the experiment.

The gradient temperature field induced by hands is the key for both the static and the dynamic manipulation of convection patterns. We thus compared the experimental thermal field and the simulated thermal field for the above different forms of convection manipulation. For the static generation of convections, we compared the thermal field between the experiment and simulation by placing one hand at one side (Figure S13a, Supporting Information), placing two hands at the left and right sides symmetrically and asymmetrically (Figure S13b, Supporting Information), placing the index finger underneath the container (Figure S13c, Supporting Information), and placing two hands and the index finger at three different positions of the container (Figure S13d,e, Supporting Information). For the dynamic control of convections, we compared the thermal field between the experiment and transient simulation by placing the hand (Figure S14a) and the index finger (Figure S14b, Supporting Information) on the right side of the container and then switching to the other side of the container. In both Figures S13 and S14 (Supporting Information), the experimental and simulated thermal fields show similar temperature distributions. The slight differences between the thermal fields observed in the experiment and shown in the simulation might be due to the conditions we used in the simulation. In the simulation, we simplified the model of hand as a slab with the size of 4 cm × 4 cm and the thickness of 1 cm and the model of the index finger as a stick with the size of 1 cm × 1 cm and the length of 4 cm. We also simplified the environment condition with uniform temperature and without air convection.

### 2.4 Demonstration of the Potential Applications of the Touchless Hand‐IR Driven Convection System

**Figure 5:**
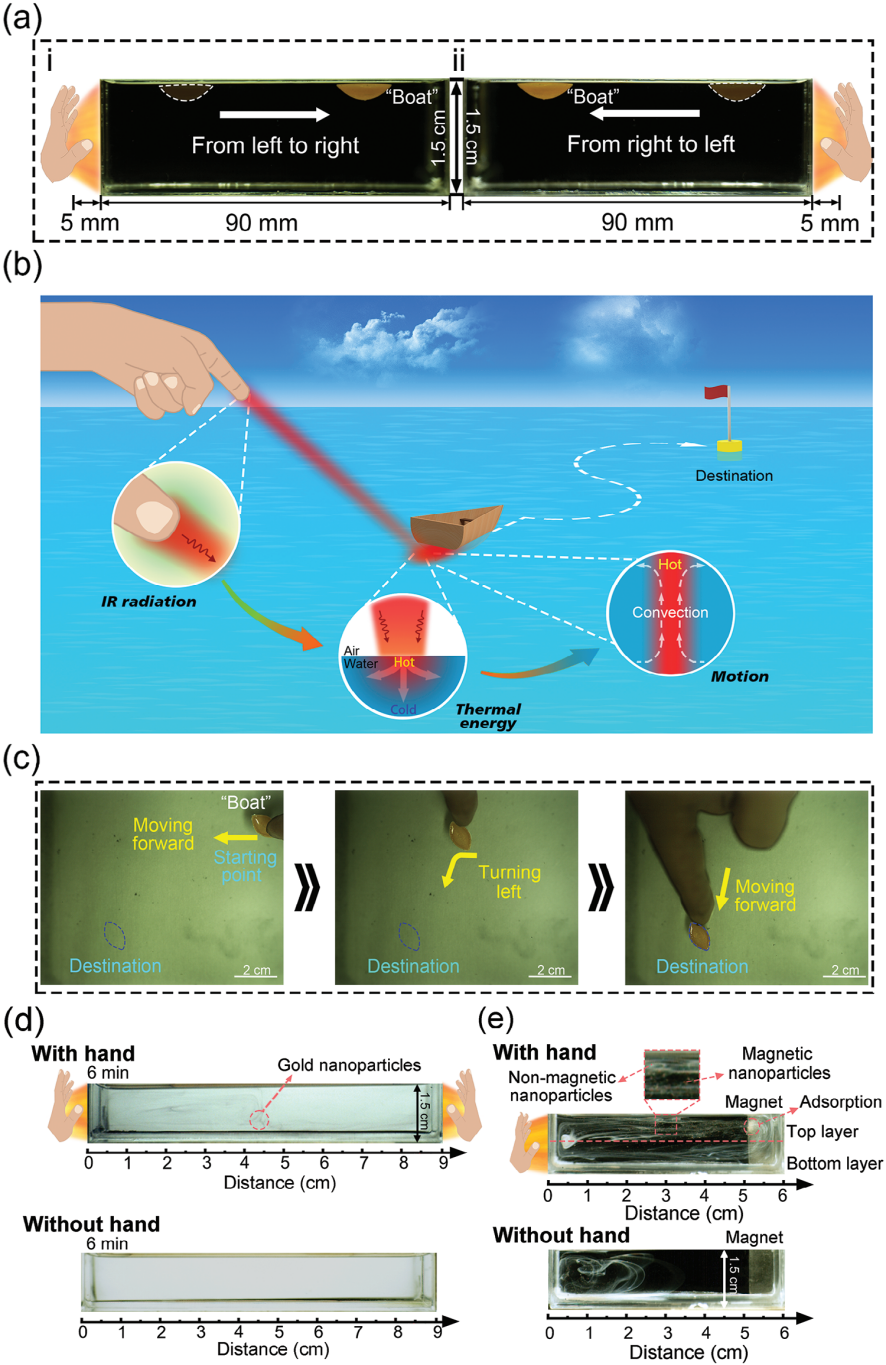
Demonstrations of the potential applications of the touchless hand‐IR driven convection system. a) The hand generated convection can induce the movement of “boat” inside the container. The “boat” sails from one side to another side when the hand is placed close to the side wall of the container. b) The localized convection can be generated near the “boat” using hand. c) The movement of the floating “boat” can be controlled with the movement of finger dynamically to sail on the liquid surface to the desired destination. d) With the hand‐generated convection, the chloroauric acid could react with ascorbic acid in 6 min and form the gold nanoparticles. e) With the hand placed on the left side of the container, the mixed PS nanoparticles could be transported to the right side of the container and be separated by the magnet. Without the hand‐generated convection, the mixed PS nanoparticles were randomly distributed in the fluid.

With the demonstration of static and dynamic control of the convections by hands, we further explored the control of the motion of a floating “boat” at the liquid surface inside a container. Although the stable and clear convective patterns require placing hand for 6 min, the respond of the convection is rapid. For the demonstrations of driving “boat”, which are transient processes, the hands can move at will and do not need to wait the stable convection formed. We first demonstrated the static control of the “boat” by placing one hand on the left side of the containers (**Figure** **5**a,i). With such placement of the hand, single clockwise convection was formed on the left side of the container. With the convection induced mass transport of fluid, the “boat” moved from the left to the right inside the container (Movie S2, Supporting Information). When we placed the hand on the right of the container the convection formed at the right side of the container with counter clockwise motion and the “boat” moved back to the left side (Figure 5a,ii).

We further explored the use of IR radiation from the finger to control the motion of the floating “boat” on the DI water surface at any direction through the control of convection (Figure 5b). A container with size of 30 cm × 30 cm × 3 cm was used in this demonstration. Such container is much larger than the containers used in the previous experiments, so the influence from the sidewalls of the container can be avoided. For a proof‐of‐concept demonstration, with the control of convection by finger, the floating “boat” first moved forward from the starting point, then turned left, and eventually arrived at the destination. As shown in Figure 5c and Movie S3 (Supporting Information), the movement of the floating “boat” followed the local fluid motion of the convection generated by finger. We further used COMSOL to simulate the temperature distribution of the liquid when a finger is placed 1 mm above the water surface (Figure S15a, Supporting Information). The simulating result of the temperature field proved that the absorption of IR radiation from the finger by water played a major role to generate the convection that provides the driving force of the floating “boat”. Since the convection was generated close to the finger, with the movement of the finger, the movement of floating “boat” can follow the change of the finger position (Figure S15b, Supporting Information).

Besides the control of the movement of a floating “boat”, we further demonstrated the potential applications of the hand‐controlled convection in sensing and separation systems. For bio/chemical sensing systems, the convection has the potential to shorten the sensing time. As a proof‐of‐concept demonstration, we ran an experiment for the rapid detection of chloroauric acid aided by the hand‐induced convection. We first prefilled 40 mL of DI water in the 9 cm‐long container and then dropped 15 µL of chloroauric acid solution (6.8 mg mL^−1^) on the left side of the container and 15 µL of ascorbic acid (3.5 mg mL^−1^) on the right side of the container. The hands were placed on both sides of the container and the distance between the hands and the container was 5 mm. We also ran a control experiment, in which the hands were not placed near the container. As shown in Figure 5d, hand‐induced convection could promote the transportation of both the chloroauric acid and the ascorbic acid to the middle of the container for the formation of the reddish gold nanoparticles in 6 min. In comparison, the gold nanoparticles cannot be observed in 6 min in the experiment without hands.

For the separation system, the convection could transport the mixtures to the target for separation by the liquid flow. We ran the following experiments to demonstrate the separation of the magnetic PS nanoparticles and the non‐magnetic PS nanoparticles that was also assisted by the hand‐induced convection. First both the initial non‐magnetic PS solution of 8.0% w/v and magnetic PS solution of 2.5% w/v were diluted with DI water to a concentration of 1.0% w/v. We then dropped 15 µL of non‐magnetic PS solution and 15 µL of the magnetic PS solution simultaneously on the left side of the 6 cm‐long container that was prefilled with 27 mL DI water. The non‐magnetic PS nanoparticles showed the white color in the fluid and the magnetic PS nanoparticles showed the reddish color in the fluid. The hand was placed on the left side of the container and the magnet was placed on the right side of the container. After the convection was generated (≈ 6 min), the top layer of convection transported the mixed non‐magnetic and magnetic PS nanoparticles to the right side of the container and the magnet could attract the magnetic PS nanoparticles (Figure 5d). The bottom layer of the convection, which flowed back to the right side of the container, thus only contained the non‐magnetic PS nanoparticles after the separation of the magnetic PS nanoparticles. In such control experiment without the hand, the mixed non‐magnetic and magnetic PS nanoparticles could not be transported to the right end of the container for the separation within the same time period (6 min).

The above demonstrations show that objects with different size levels could be transported by the hand‐IR controlled convection system. Despite that the response time is a few seconds, the system could still be leveraged in some possible applications. For example, in the field of polymerase chain reaction (PCR), the temperature difference‐induced convection could reduce the amplification time of the PCR to <30 min, which was only 1/3 to 1/2 of the normal PCR amplification time.^[^ ^48^, ^49^ ^]^

## 3 Discussion

In summary, we demonstrated the use of hand, which is a nature light source without the need of electricity, for the generation and manipulation of the convection with comparable performance to those reported in literature that were powered by engineering light sources (Table S1, Supporting Information). The absorption of the IR radiation emitted from hand results in the generation of the temperature difference inside the fluid for the formation of the convection. The experimental results and numerical calculations demonstrated that both the velocity and the lateral size of the convection increase with the increase of the depth of the water and the decrease of the distance between the hands and the container. Complex convections, with both symmetric and asymmetric patterns, could be formed along various directions by controlling the positions of the hands. Besides generating the convection statically, the convection can also be dynamically formed and controlled by changing of the positions of the hand or finger. By taking the advantage of the dynamic control of the localized convections, the sustainable flexible control of the floating “boat” at the liquid‐air surface was further demonstrated in this study. The findings in this work show the feasibility of controlling the fluidic convections at will by integrating hands as sustainable and flexible IR energy sources into the system, which may further expand the possible application scope of the convection in low‐cost sensing and separation systems.

## 4 Experimental Section

### Generation and Observation of the Convection

The containers were made of quartz with the same cross‐section area of 3 cm × 3 cm but with different lateral lengths (3 cm, 6 cm, and 9 cm). The PS nanospheres with sulfate functional groups were purchased commercially from Invitrogen Trading (Shanghai) Co., Ltd. The average diameter of PS particles is about 206 nm. The initial PS solution of 8.0% w/v was diluted with DI water to a concentration of 1.0% w/v. Before the experiments, the 3 cm, 6 cm, and 9 cm containers were prefilled with 13 mL, 27 mL, and 40 mL DI water, respectively. A planar LED light source (Juhua Vision Technology Co., Ltd) with an illumination area of 13.8 cm × 7.3 cm was used as the background light for the capture of convection images. The hands and the fingers are placed on different sides of the containers to control the convections. About 8 µL of the above diluted PS solution was slowly dropped into the DI water for tracing the flow of the fluid. In order to isolate the IR radiation from the other part of the hand, the hand was wrapped by aluminum foil with an emitting window of 4 cm × 4 cm, which could cover the sidewall of the container. The temperature distribution of the DI water and the containers was measured by an IR camera (FLIR T640, Teledyne FLIR LLC). The high‐speed camera (S‐VIT LS, AOS Technologies AG) was used to record the formation and final state of the convection with a recording speed of 8 frames per second.

### The Measurement of the Velocity and the Lateral Size of the Convection

We first placed the hand next to the container. The stable convection was formed after 6 min. Then one drop (≈ 8 µL) of diluted PS solution was slowly dropped into the liquid to trace the flow. A digital camera (Canon EOS 7D) was used to record the formation of the convection with a recording speed of 30 frames per second. In all experiments, we picked PS particles at one specific position (≈ 0.12 cm to the liquid/air surface and ≈ 2 cm to the left side of the container) and measured the moving distance of these PS particles in 20 s. The average velocity of the convection can thus be calculated. The lateral size of the convection was obtained by measuring the length of the convection patterns along the lateral direction.

### Convection Controlled Movement of Floating “Boat” by IR Radiation Emitted from Hand

In this demonstration, we used the hands and the fingers as the IR energy sources to drive a “boat” floating at the surface of water. The “boat” (15 mm × 8 mm × 5 mm) was made of polylactic acid (PLA) by 3D printing. The static control of the boat was demonstrated by placing one hand close to the side wall of the quartz container (9 cm × 3 cm × 3 cm) that was prefilled with 80 mL DI water. The high‐speed camera was placed in front of the container to record the movement of the floating “boat”. A polymethyl methacrylate (PMMA) container with size of 30 cm × 30 cm × 3 cm was used in the dynamic control experiment, and the floating “boat” was initially placed in the center of the container. The finger was placed close to the liquid‐air interface without touching the water and the floating “boat”. The moving of the “boat” was recorded by the digital camera and the high‐speed camera, which was placed on the side and the top of the container, respectively. By manipulating the pointing direction and the motion of the finger, the movement of the floating “boat” could be controlled.

## Conflict of Interest

The authors declare no conflict of interest.

## Author contributions

H.Z. and Z.L. contributed equally to this work. T.D, W.S., S.A., H.Z., and Z.L. designed research. H.Z., L.Z., Q.S., R.Y., W.C., Y.Z., M.J., and C.G. performed fabrication, testing, and characterizations. All authors discussed and analyzed the results and contributed to the writing of the paper.

## Supporting information

Supporting Information

Supplemental Movie 1

Supplemental Movie 2

Supplemental Movie 3

## Data Availability

The data that support the findings of this study are available from the corresponding author upon reasonable request.
