# Supplementary material for: Manipulation of Convection Using Infrared Light Emitted from Human Hands
Source: Adv Sci (Weinh). 2024 Jan 18;11(12):2307020. doi: 10.1002/advs.202307020 (PMC10966531; doi:10.1002/advs.202307020)
Supplement: Supplementary file 1 — Supporting Information [file ADVS-11-2307020-s003.pdf]

ADVANCED SCIENCE
Open Access

Supporting Information

for *Adv. Sci.*, DOI 10.1002/advs.202307020

Manipulation of Convection Using Infrared Light Emitted from Human Hands

Hanrui Zhu, Zhen Luo, Lifu Zhang, Qingchen Shen, Runheng Yang, Weizheng Cheng, Yingyue Zhang, Modi Jiang, Chunzhi Guo, Benwei Fu, Chengyi Song, Peng Tao, Shun An, Wen Shang* and Tao Deng**

Supporting Information

Manipulation of Convection Using Infrared Light Emitted from Human Hands

Hanrui Zhu†, Zhen Luo†, Lifu Zhang, Qingchen Shen, Runheng Yang, Weizheng Cheng, Yingyue Zhang, Modi Jiang, Chunzhi Guo, Benwei Fu, Chengyi Song, Peng Tao, Shun An, Wen Shang*, Tao Deng**

H. Zhu, Z. Luo, L. Zhang, Q. Shen, R. Yang, W. Cheng, Y. Zhang, M. Jiang, B. Fu, C. Song, P. Tao, S. An, W. Shang, T. Deng

State Key Laboratory of Metal Matrix Composites, School of Materials Science and Engineering, Shanghai Jiao Tong University, Shanghai, 200240, P. R. China.

*Corresponding author. Email: anshun@sjtu.edu.cn (S.A.); shangwen@sjtu.edu.cn (W.S.); dengtao@sjtu.edu.cn (T.D.)

L. Zhang

Department of Materials Science and Engineering, Rensselaer Polytechnic Institute, NY, 12180-3590, USA.

Q. Shen

Yusuf Hamied Department of Chemistry, University of Cambridge, Cambridge, CB2 1EW, UK.

C. Guo

School of Electronic Information and Electrical Engineering, Shanghai Jiao Tong University, Shanghai, 200240, P. R. China.

T. Deng

Shanghai Key Laboratory of Hydrogen Science, School of Materials Science and Engineering, Shanghai Jiao Tong University, Shanghai, 200240, P. R. China.

[†] These authors contributed equally to this work.

This PDF file includes:

Supplementary text
Supplementary Table S1
Supplementary Figures S1 to S11
Legends for Movies S1 to S3
Supplementary References

Other Supplementary Materials for this manuscript include the following:

Supplementary Movies S1 to S3

Supplementary Text

Section 1. Calculation of the input thermal energy for the experiments.

Since the temperature of water has been in equilibrium with that of the environment, the human hand is the only heat source in this system. The input thermal energy is thus from the IR radiation of the hand while the hand is not in contact with the container and the boat.

The area of the planar IR energy source of the hand is 4 cm * 4 cm. The net input thermal energy of the container equals to the received thermal radiation power minus the thermal radiation power from itself. According to the above analysis, the net input thermal energy can be given as the following equation:

$$Q_{net} = Q_{amb} + Q_{hand} - Q_{quartz} \tag{S1}$$

where Q_{amb} and Q_{hand} are received thermal radiation power from ambient and hand, respectively, Q_{quartz} is the thermal radiation power of the sidewall. Based on the Stefan–Boltzmann law, Q_{amb}, Q_{quartz} and Q_{hand} can be described using following equations:

$$Q_{amb} = A_{quartz}\varepsilon_{quartz}\varepsilon_{amb}\sigma T_{amb}^4(1 - \frac{F_{12}A_{hand}}{A_{quartz}}) \tag{S2}$$

$$Q_{quartz} = A_{quartz}\varepsilon_{quartz}\sigma T_{quartz}^4 \tag{S3}$$

$$Q_{hand} = A_{quartz}\varepsilon_{quartz}\frac{F_{12}A_{hand}}{A_{quartz}}\varepsilon_{hand}\sigma T_{hand}^4 \tag{S4}$$

where σ is the Stefan-Boltzmann constant that is 5.67 * 10^{-8} W (m^2 K^4)$^{-1}$, T_{amb}, T_{hand} and T_{quartz} are the temperature of ambient, hand, and container sidewall that are measured by the IR camera, respectively; A_{hand} and A_{quartz} are the area of the hand and the container sidewall, respectively; F_{12} is the view factor that is used to

describe the fraction of energy leaving from hand and reaches the sidewalls; ε_{hand} is the emissivity of the hand that is about 0.98; the ε_{amb} and ε_{quartz} are the emissivity of ambient and the absorptivity of the sidewall, respectively. The absorptivity of the sidewall can be measured by Fourier transform infrared (FTIR) spectrometer (Figure S3 (a)). The view factor in Equation S4 is only related to the shape and distance of the hand and the sidewalls. The size of the sidewall was 3 cm * 3 cm. We used an aluminum (Al) foil with an opening of 4 cm * 4 cm for the emission window and blocked the IR radiation from the other parts of the hand. In this case, the size of hand is 4 cm * 4 cm and the view factor decreases with increasing distance between the hand and the sidewalls. The decreasing of view factor thus causes the less net input thermal energy. Similarly, for demonstrations of placing the finger under the bottom of the container, the finger can be considered as a linear heat source. The Equation S1 to Equation S3 is still valid for this case, and only the Equation S4 needs to be modified. If we define the left side of the container as the origin and the lateral direction as x direction, we can calculate the distribution of the received irradiance on the bottom surface from the hand:

$$E_{hand}(x) = \frac{d}{2\sqrt{(x-x_{hand})^2+y^2}} \cdot \sigma T_{hand}^4 \tag{S5}$$

where $E_{hand}(x)$ is the received irradiance at the position x, d is the diameter of the finger, x_{hand} is the distance between the finger and the origin along the x direction, and y is the distance between the finger and the bottom surface. With the known distribution of the received irradiance, the received IR radiation power can be given as the following equation:

$$Q_{hand} = \int_0^{x_{right}} E_{hand}(x)dx \quad \text{(S6)}$$

where x_{right} is the position of the right end of the container. With the above equations, the net input thermal energy of the containers can be numerically calculated for both the experiments using hands and using fingers as the IR light sources.

Section 2. COMSOL Multiphysics simulation of the generation of the convection.

The generation and the control of the convection by human hands were simulated by finite element method (FEM) using COMSOL Multiphysics. Since the Al foil with a square window was used to block the radiation from the other part of the hands, we thus assume the IR light source from the hand as square light source. The structural parameters (depth, thickness of the sidewall, et al.) of the containers and the DI water are set according to the experimental condition. The ambient temperature is set as 20 °C for the following calculation. The temperature of the hands and fingers are set to a constant value of 37 °C. The boundary condition of the sidewall is set as the external natural convection. The global mass and momentum balances for nonisothermal flow are coupled to an energy balance, where heat transport occurs through convection and radiation. According to the experimental data, we can calculate the average absorptivity of the quartz as the following equation:

$$\varepsilon_{ave} = \frac{\int \varepsilon(\lambda)\frac{2\pi hc^2}{\lambda^5}\cdot\frac{1}{e^{\frac{hc}{\lambda kT}}-1}d\lambda}{\int \frac{2\pi hc^2}{\lambda^5}\cdot\frac{1}{e^{\frac{hc}{\lambda kT}}-1}d\lambda} \quad \text{(S7)}$$

where h is Planck's constant, c is the speed of light in vacuum, k is Boltzmann constant, λ is the wavelength of the radiation, T is the absolute temperature of the

blackbody given in Kelvin, and $\varepsilon(\lambda)$ is the function relationship between absorptivity and wavelength. From the Equation S7, the average absorptivity of the quartz is related to temperature of the quartz. After calculating ε_{ave} from 20 °C to 40 °C, the numerical fitness is used to describe the relationship between ε_{ave} and the temperature of the quartz. We used a physics-controlled mesh to obtain the boundary layers at the water-glass interface. By solving the Equations 1 in the manuscript, the velocity field and the temperature field of the convection could be obtained.

Section 3. The Boussinesq approximation for the Navier-Stokes equations.

The Boussinesq approximation is a way to solve nonisothermal flow and can directly show the relationship of the temperature change and the velocity field. To derive Equation 1 in the main text, we start with the original Navier-Stokes equation that used for describing the momentum of fluid, and it can be given as:

$$\rho\frac{\partial \boldsymbol{u}}{\partial t} + \rho\boldsymbol{u}\cdot\nabla(\boldsymbol{u}) = -\nabla p_{NS} + \nabla\cdot\left(\mu\left(\nabla\boldsymbol{u} + \nabla\boldsymbol{u}^{\mathrm{T}}\right) - \frac{2}{3}\mu\left(\nabla\cdot\boldsymbol{u}\right)\mathbf{I}\right) + \boldsymbol{F}_{NS} \quad \text{(S8)}$$

where $\boldsymbol{u}$, p_{NS} and $\boldsymbol{F}_{NS}$ are the velocity vector, the pressure, and the body force, and ρ is the density of the fluid. $\boldsymbol{F}_{NS}$ equals to $\rho\boldsymbol{g}$ in this study. Using the Boussinesq approximation requires that the temperature-induced density variation $\Delta\rho$ of the fluid is much less than the initial density of the fluid ρ_0 at the initial temperature T_0. In this study, the maximum density variation $\Delta\rho$ is around 0.0012 g/cm3 and the initial density of the fluid ρ_0 is around 0.998 g/cm3, which is applicable to the requirement of the Boussinesq approximation.

The Boussinesq approximation states that the density variation is only important

in the buoyancy term, which is the variation of the body force, and can be neglected in the rest of the equation. The Equation S8 can be changed into:

$$\rho_0 \frac{\partial \boldsymbol{u}}{\partial t} + \rho_0 \boldsymbol{u} \cdot \nabla(\boldsymbol{u}) =- \nabla p_{NS} + \nabla \cdot \left(\mu\left(\nabla \boldsymbol{u} + \nabla \boldsymbol{u}^{\mathrm{T}}\right) - \frac{2}{3}\mu(\nabla \cdot \boldsymbol{u})\mathbf{I}\right) + \rho \boldsymbol{g}. \qquad \text{(S9)}$$

In this study, $\boldsymbol{F}_{NS} = \rho \boldsymbol{g}$ is the gravitational body force along the vertical direction z. The ρ can be rewritten as $(\rho - \rho_0 + \rho_0)$, which $(\rho - \rho_0)$ is the variation of density of the fluid $\Delta\rho$ when the temperature changes from T_0 to T. Based on the thermal expansion theory, the relationship between the variation of temperature and density can be given as:

$$\Delta\rho =- \rho_0 \beta \Delta T \qquad \text{(S10)}$$

where β is the coefficient of thermal expansion of the fluid at T_0 and ΔT is the variation of the temperature. According the previous work of the Boussinesq approximation by D. J. Tritton[1], the Equation S9 can be thus given as following format:

$$\rho_0 \frac{\partial \boldsymbol{u}}{\partial t} + \rho_0 \boldsymbol{u} \cdot \nabla(\boldsymbol{u}) =- \nabla(p_{NS} + \rho_0 g z) + \nabla \cdot \left(\mu\left(\nabla \boldsymbol{u} + \nabla \boldsymbol{u}^{\mathrm{T}}\right) - \frac{2}{3}\mu(\nabla \cdot \boldsymbol{u})\mathbf{I}\right) - \rho_0 \beta (T - T_0) \boldsymbol{g} \qquad \text{(S11)}$$

In the Equation S11, we defined $(p_{NS} + \rho_0 g z)$ as the approximated pressure p and $- \rho_0 \beta (T - T_0) \boldsymbol{g}$ as the approximated body force $\boldsymbol{F}$, which is the buoyancy term. Finally, the original equation that used for describing the motion of the fluid can be given as following format under the Boussinesq approximation:

$$\rho_0 \frac{\partial \boldsymbol{u}}{\partial t} + \rho_0 \boldsymbol{u} \cdot \nabla(\boldsymbol{u}) =- \nabla p + \nabla \cdot \left(\mu\left(\nabla \boldsymbol{u} + \nabla \boldsymbol{u}^{\mathrm{T}}\right) - \frac{2}{3}\mu(\nabla \cdot \boldsymbol{u})\mathbf{I}\right) + \boldsymbol{F} \qquad \text{(S12)}$$

The Equation S12 (Equation 1 in the main text) shows that the variation of temperature can induce the change of the density and the expansion of fluid, which

leads to the flow of fluid from the hot side to the cold side.

Section 4. The calculation of the penetration depth of the water.

Water has high absorptivity in the far-IR wavelength range due to the vibrational energy of the molecules[2]. In order to accurately evaluate the absorption capacity of the water in the IR wavelengths, the penetration depth is used to characterize how deeply IR light can transport into the water. The penetration depth is defined as the depth at which the intensity of the radiation inside the medium falls to 1/e of its original value. The penetration depth can be calculated using the following equation:

$$d_p = \frac{-\lambda_0}{4\pi \cdot Im\sqrt{\varepsilon}} \tag{S13}$$

where d_p is the penetration depth of the water, λ_0 is the free space wavelength of the IR and ε is the complex dielectric permittivity with the real component $\varepsilon^{'}$ and imaginary component $\varepsilon^{''}$. With the known complex dielectric permittivity of the water[3], the penetration depth of the water can be easily solved (Figure S3(c)). Since the IR radiation emitted by human hands mainly is in the range of 4 μm to 16 μm, the d_p is calculated to be less than 100 μm. Such a small value indicate that the IR light emitted from the hand can only penetrated a very short distance inside the water.

Section 5. Characterization of accuracy and sensitivity of fluid velocity changes with energy.

The accuracy is generally used to evaluate the consistency between the simulated and the experimental results. The root mean square errors (RMSE) of the numerical

results with respect to the experimental data is used to quantitatively analyze the accuracy[4, 5]. The RMSE could be calculated by following equation:

$$RMSE = \sqrt{\frac{\sum_{i=1}^{n}(x_i - x_s)^2}{n}} \qquad (S14)$$

Where x_i and x_s are the experimental and simulation result of the fluid velocity and n is the number of repeated experiments.

The sensitivity of fluid velocity changes with energy is used to characterize the relationship between fluid velocity and input energy. The gradient of the relationship between input energy and fluid velocity is used to represent sensitivity[6]. Here we changed the input energy of the convection system by placing the hand at different distances to the container and repeated 5 times of the generation of the convection under the same input energy to characterize the accuracy and sensitivity.

Table S1. The performance parameters of the movement control in our work and previous work.

Authors	Energy Source	Power density	Whether electricity powered	Velocity	Response time
Roxworthy, et al. [7]	NIR laser	~2.58*10^8 mW/cm^2	Yes	>1*10^{-3} mm/s	—
Seshadri, et al. [8]	White light source	214 mW/cm^2	Yes	3 mm/s	<5 s
Winterer, et al. [9]	NIR laser	~8.72*10^8 mW/cm^2	Yes	1.27*10^{-3} mm/s	<3 s
Deng, et al. [10]	NIR laser	6.6*10^4 mW/cm^2	Yes	0.073 mm/s	<5 s
Chai, et al. [11]	Heat plate	—	Yes	—	—
Rodrigo, et al. [12]	Laser	~0.54*10^8 mW/cm^2	Yes	0.052 mm/s	—
Jin, et al. [13]	NIR laser	~0.76*10^8 mW/cm^2	Yes	1.67*10^{-3} mm/s	<6 s
Dinh, et al. [14]	NIR laser	600 mW/cm^2	Yes	0.1 mm/s	<3 s
Our work	Hand	51 mW/cm^2	No	0.31 mm/s	~5 s

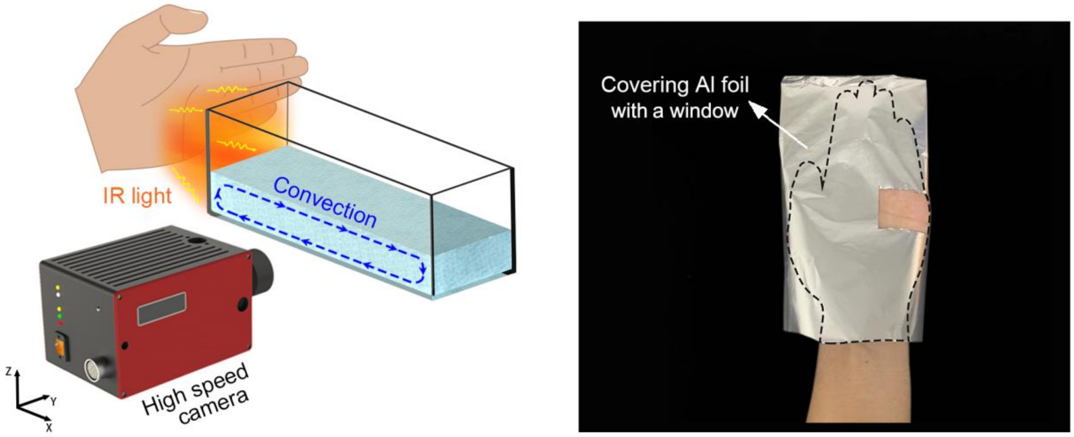

Figure S1. The setup for the experiment. (a) Experimental setup for the generation of the convection by hand. **(b)** The hand is covered with Al foil with an IR window.

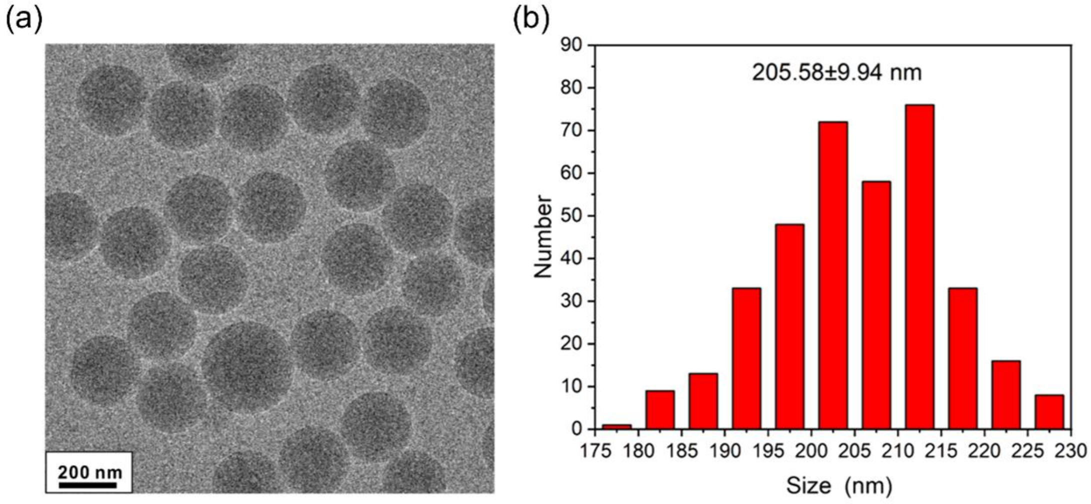

Figure S2. Characterization of the PS nanoparticles. (a) TEM image of PS nanoparticles that are used for tracing the convection. **(b)** The size distribution of PS nanoparticles.

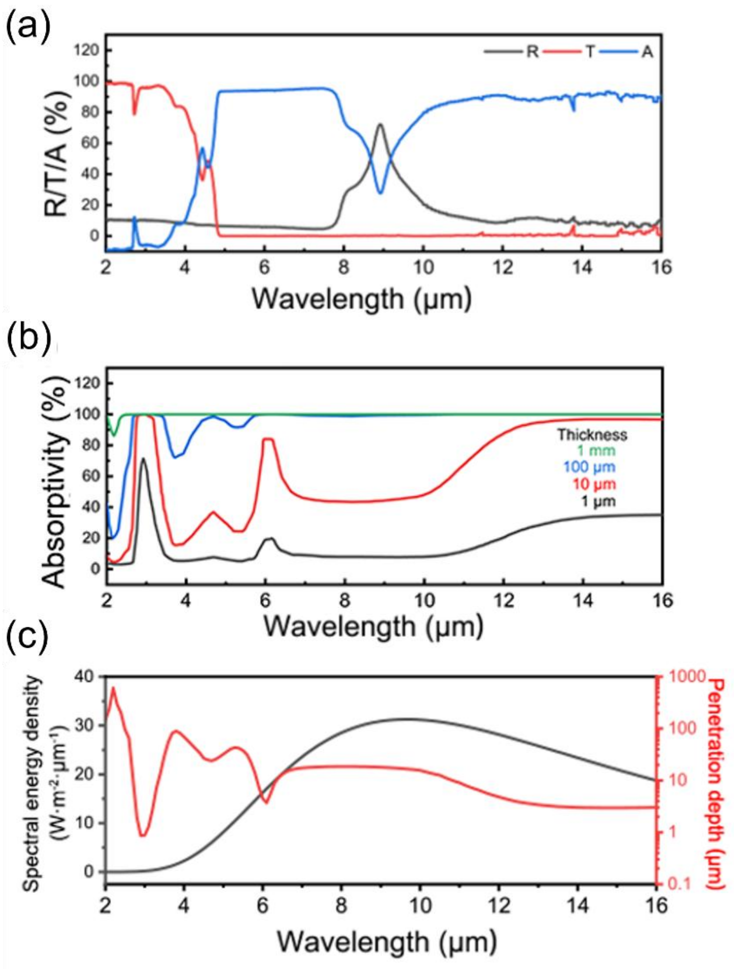

Figure S3. The IR spectra of the quartz and the water used in the experiments. (a) The IR spectrum of reflectivity, transmissivity, and absorptivity of quartz with a thickness of 1mm. **(b)** Absorptivity spectra of slabs of water of varying thicknesses[15]. **(c)** The calculated penetration depth (Supplementary Text, Section 4) of the water and the emission spectrum of hands are shown in the red line and the black line, respectively.

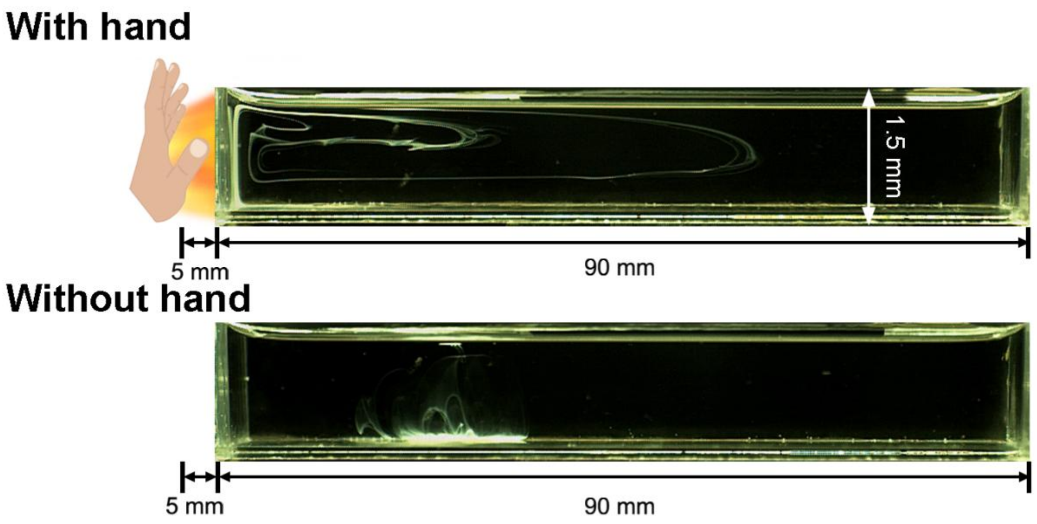

Figure S4. The generation of convections with hand and without hand.

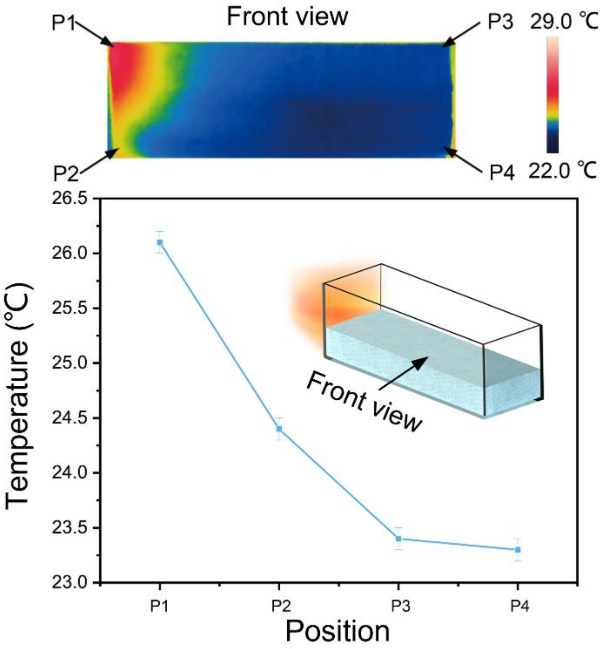

Figure S5. The measured average temperature and IR image of the front side of the container when hand is on the left side.

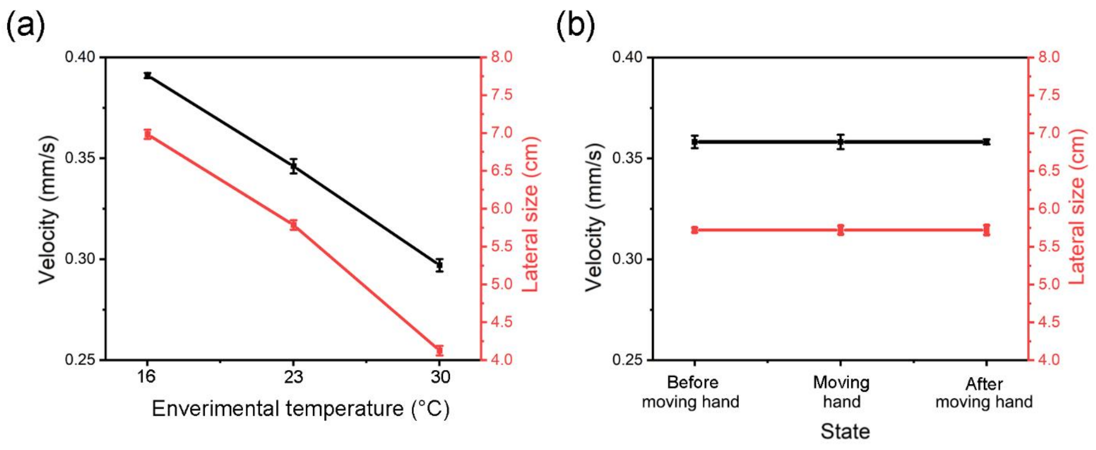

Figure S6. The effect of the environmental temperature and the movements of extra IR light sources on the convection. (a) The lateral size (red line) and the velocity (black line) of the convection under different environmental temperature. **(b)** The lateral size (red line) and the velocity (black line) of the convection when moving the other hand behind the container with a speed of 1.2 cm/s. The error bars represent the standard deviation (SD) of the mean (n = 5).

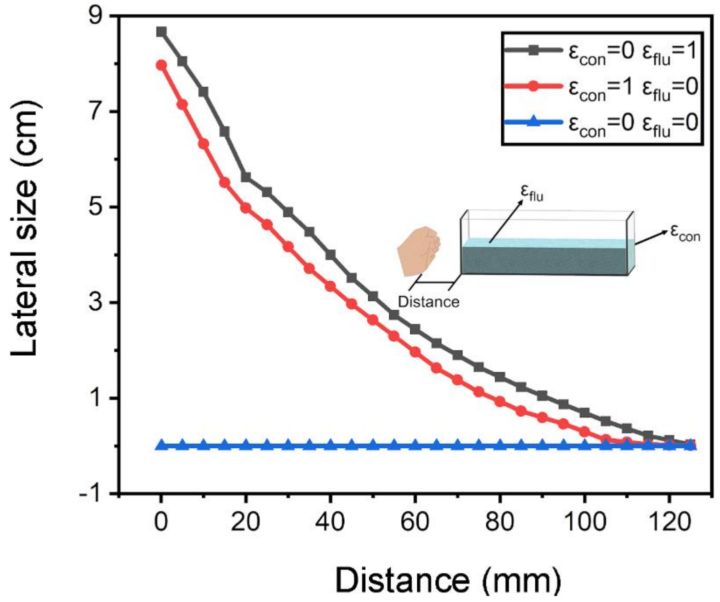

Figure S7. The lateral size of the convection generated in different scenarios. The change of the lateral size of the convection with the change of the distance between the hand and the container in three different scenarios.

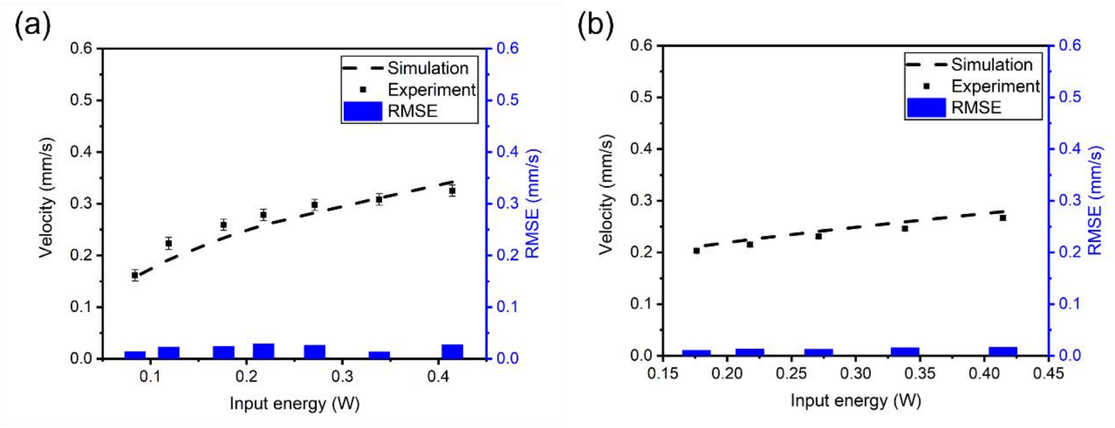

Figure S8. The change of fluid velocity with the change of the input energy. (a) The change of fluid velocity with the change of the input energy of the unconstrained convection. **(b)** The change of fluid velocity of with the change of the input energy of the constrained convection. The error bars represent the standard deviation (SD) of the mean (n = 5).

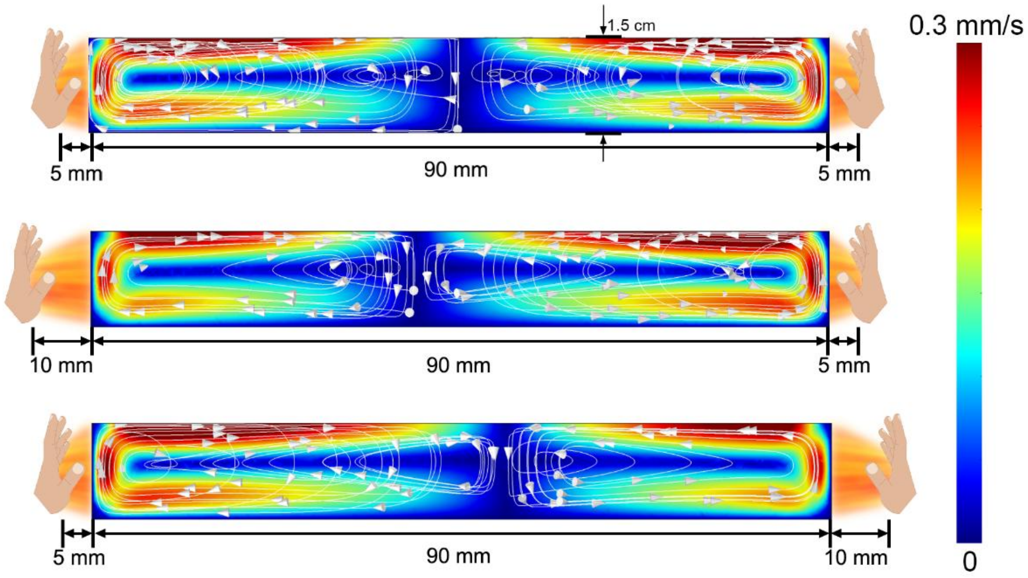

Figure S9. The simulation results of velocity field. (a) Simulation result of placing two hands symmetrically on the left and right sides of the container. **(b, c)** Simulation result of placing two hands asymmetrically on the left and right sides of the container.

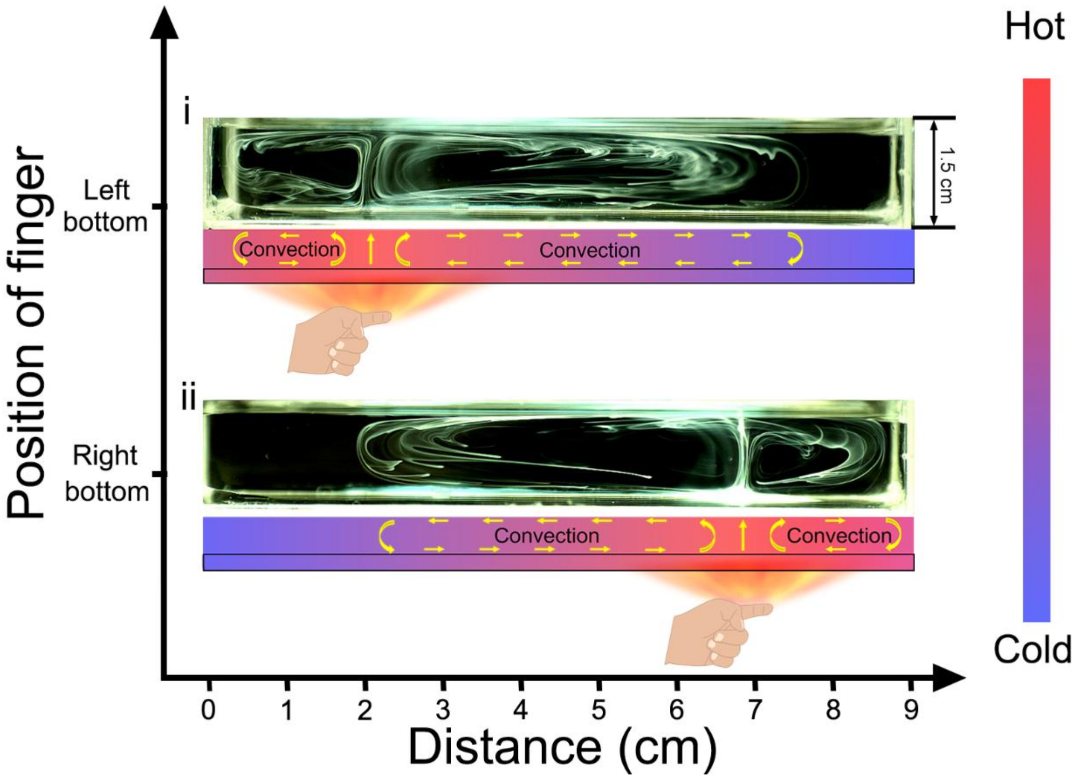

Figure S10. The generation of the convection by placing a finger at the different positions (i, ii) of the bottom of the container.

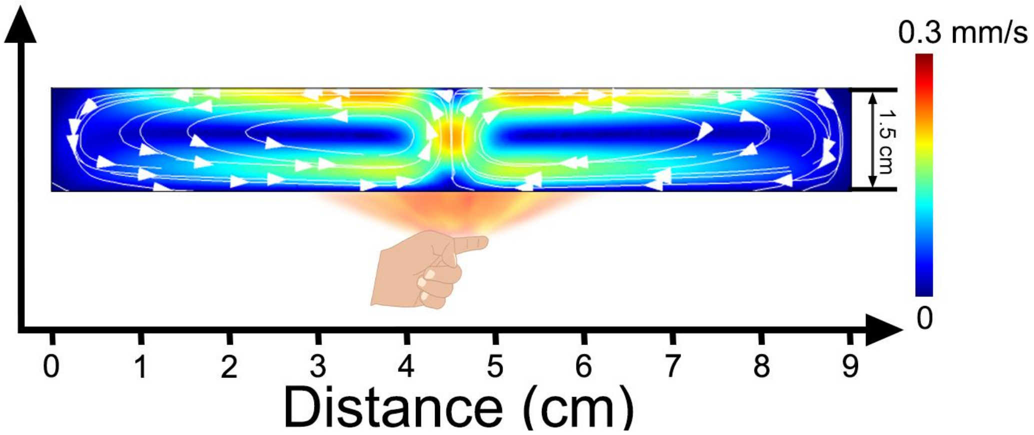

Figure S11. The simulation results of placing fingers at the middle of the bottom of the container.

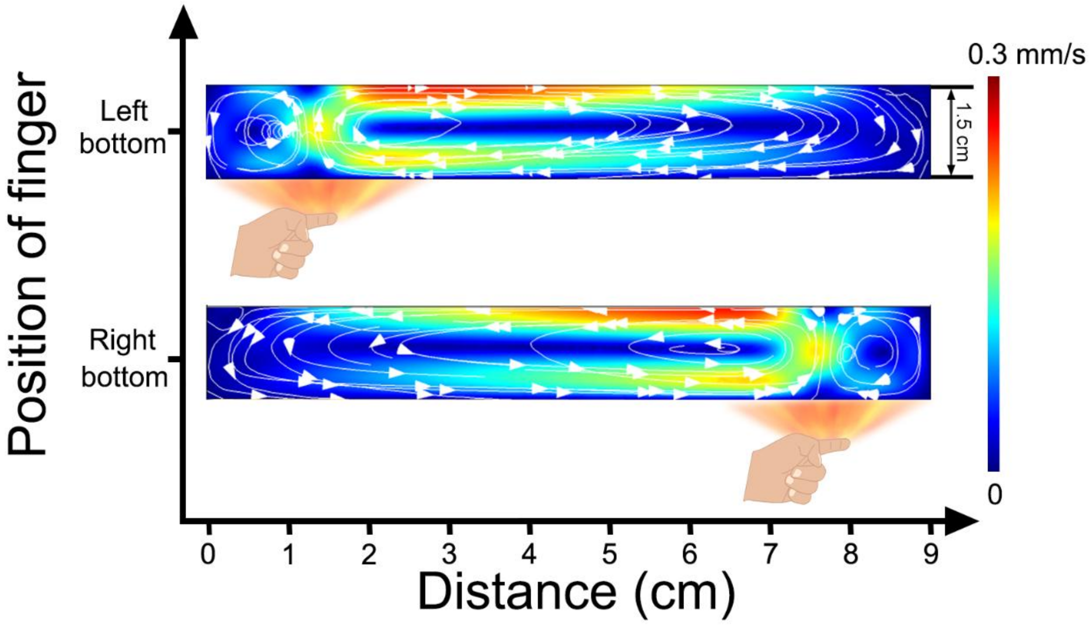

Figure S12. The simulation results of velocity field within water by placing fingers at different places (i, ii) to control the convections.

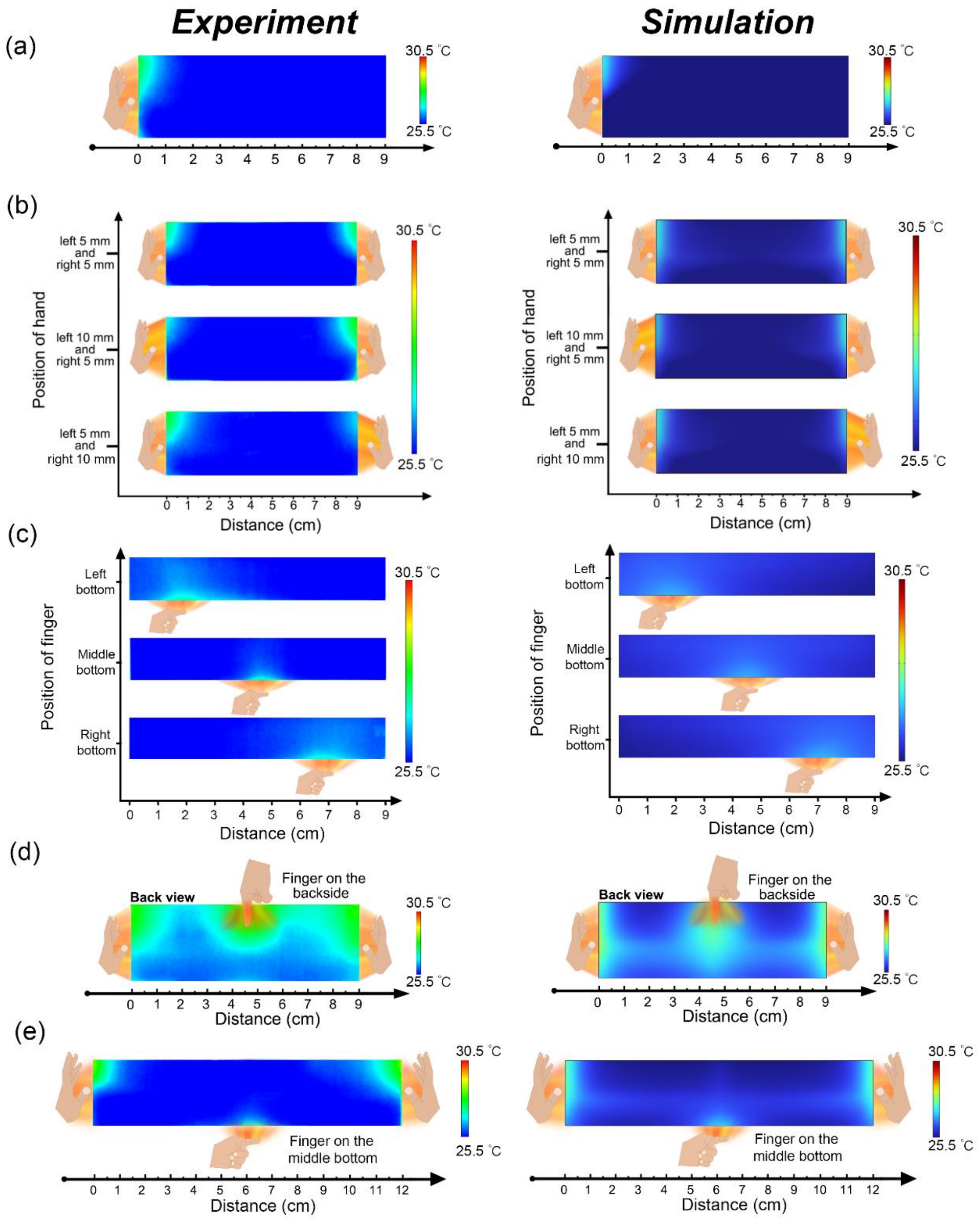

Figure S13. The comparison of thermal field of static control of the convection. (a) The experimental and the simulated results of the thermal field by placing the hand on the left side of the container. **(b)** The experimental and the simulated results of the thermal field by placing hands on both the left and right sides of the container symmetrically and asymmetrically. **(c)** The experimental and the simulated results of

the thermal field by placing the index finger underneath the left, middle, and right sides of the container. **(d)** The experimental and the simulated results of the thermal field by placing hands and the index finger on the left, right, and back of the container respectively. **(e)** The experimental and the simulated results of the thermal field by placing hands and the index finger on the left, right, and bottom of the container respectively.

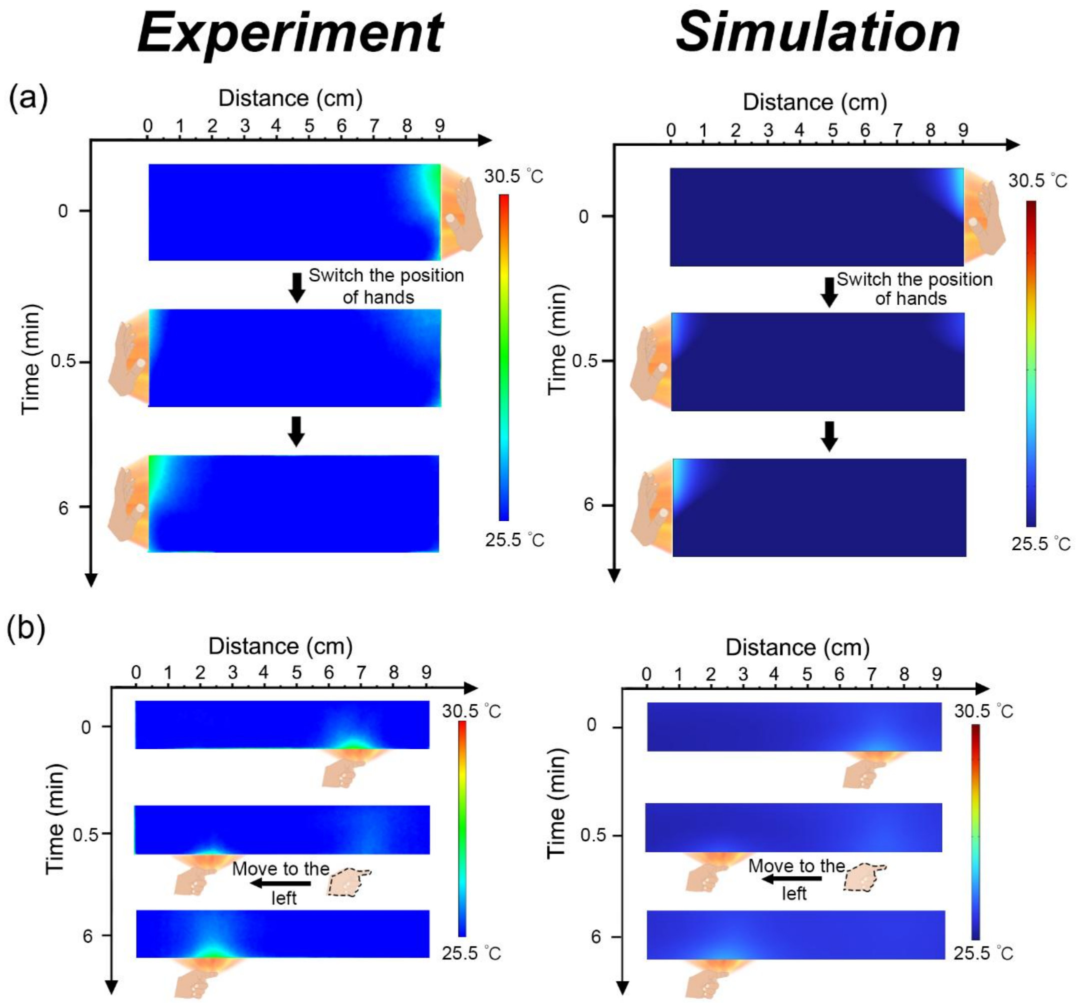

Figure S14. The comparison of thermal field of dynamic control of the convection. (a) The experimental and the simulated results of the thermal field by placing the hand on the right side of the container for 6 minutes and then switching to the left side of the container. **(b)** The experimental and the simulated results of the thermal field by placing the index finger underneath the right side of the container for 6 minutes and then moving underneath the left side of the container.

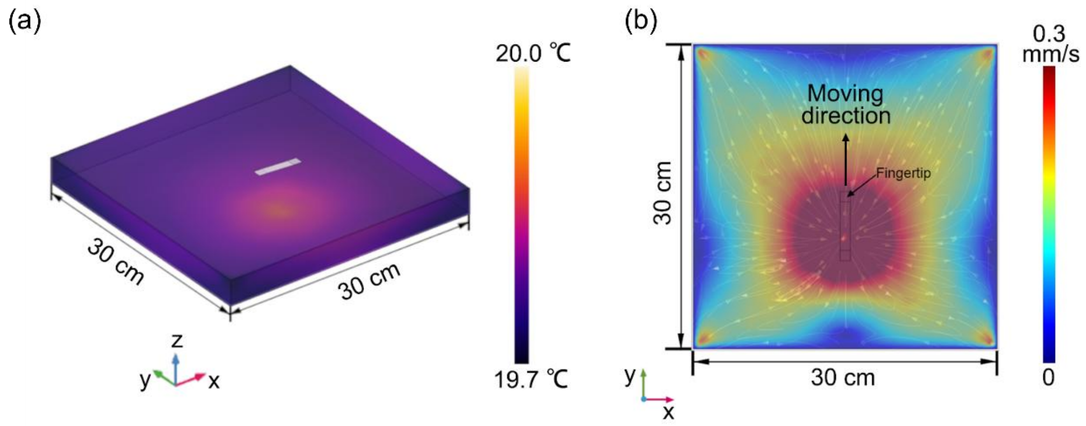

Figure S15. The simulation results of dynamic control of the floating "boat". (a) The simulated temperature field of the container with the size of 30 cm * 30 cm * 3 cm. **(b)** The simulated velocity field of the convection.

Supplementary Movie S1. Generation of the convection by hand.

Supplementary Movie S2. Static control of a floating "boat" moving at the liquid/air surface by the IR light emitted from the hand.

Supplementary Movie S3. Dynamic control of the floating “boat” by fingers.

Supplementary References

1. D. J. Tritton, *Physical Fluid Dynamics* Oxford University Press, New York, The United State 1988.
2. P. N. Prasad, *Introduction to biophotonics*, John Wiley & Sons, New Jersey, The United State 2004.
3. G. M. Hale, M. R. Querry, Optical constants of water in the 200-nm to 200-μm wavelength region. *Appl. Opt.* **1973**, *12*, 555-563.
4. T. Ma, J. Zhao, Z. Li, Mathematical modelling and sensitivity analysis of solar photovoltaic panel integrated with phase change material, *Appl. Energy*, **2018**, *228*, 1147-1158.
5. H. Jafari, M. Goharkhah, Application of electromagnets for forced convective heat transfer enhancement of magnetic fluids, *Int. J. Therm. Sci.* **2020**, *157*, 106495.
6. S. Hussain, K. Rasheed, A. Ali, N. Vrinceanu, A. Alshehri, Z. Shah, A sensitivity analysis of MHD nanofluid flow across an exponentially stretched surface with non-uniform heat flux by response surface methodology. *Sci. Rep.* **2022**, *12*, 18523.
7. B. J. Roxworthy, A. M. Bhuiya, S. P. Vanka, K. C. Toussaint Jr, Understanding and controlling plasmon-induced convection. *Nat. Commun.* **2014**, *5*, 3173.
8. S. Seshadri, L. F. Gockowski, J. Lee, M. Sroda, M. E. Helgeson, J. R. de Alaniz, M. T. Valentine, Self-regulating photochemical Rayleigh-Bénard convection using a highly-absorbing organic photoswitch. *Nat. Commun.* **2020**, *11*, 2599.
9. F. Winterer, C. M. Maier, C. Pernpeintner, T. Lohmuller, Optofluidic transport and manipulation of plasmonic nanoparticles by thermocapillary convection. *Soft Matter* **2018**, *14*, 628-634.
10. Z. Y. Deng, F. Z. Mou, S. W. Tang, L. L. Xu, M. Lou, J. G. Guan, Swarming and collective migration of micromotors under near infrared light. *Appl. Mater. Today* **2018**, *13*, 45-53.

11. Z. Chai, A. Korkmaz, C. Yilmaz, A. A. Busnaina, High-rate printing of micro/nanoscale patterns using interfacial convective assembly. *Adv. Mater.* **2020**, *32*, 2000747.

12. J. A. Rodrigo, M. Angulo, T. Alieva, Tailored optical propulsion forces for controlled transport of resonant gold nanoparticles and associated thermal convective fluid flows. *Light Sci. Appl.* **2020**, *9*, 181

13. C. M. Jin, W. Lee, D. Kim, T. Kang, I. Choi, Photothermal convection lithography for rapid and direct assembly of colloidal plasmonic nanoparticles on generic substrates. *Small* **2018**, *14*, 1803055.

14. N. Dinh, R. Luo, M. T. A. Christine, W. N. Lin, W. Shih, J. C. Goh, C. Chen, Effective Light Directed Assembly of Building Blocks with Microscale Control. *Small* **2017**, *13*, 1700684.

15. M. Vollmer, K. P. Mollmann, *Infrared Thermal Imaging: Fundamentals, Research and Applications*, Wiley, Weinheim, Germany 2017.
